# Influence of the appendicular skeletal muscle mass index on the bone mineral density of postmenopausal women

**DOI:** 10.1186/s12891-021-04748-x

**Published:** 2021-10-09

**Authors:** Geise Ferreira da Cruz, Tatiana Mion Lunz, Tatielle Rocha de Jesus, Mariana Braga Costa, Camila Vilarinho Vidigal, Ben-Hur Albergaria, Jose Luiz Marques-Rocha, Valdete Regina Guandalini

**Affiliations:** 1 Postgraduate Program in Nutrition and Health, Federal University of Espirito Santo, Marechal Campos, avenue, 1468 – Maruípe, Vitória, Espírito Santo CEP: 29040-090 Brazil; 2 Department of Integrated Education, Federal University of Espirito Santo, Marechal Campos, avenue, 1468 – Maruípe, Vitória, Espírito Santo CEP: 29040-090 Brazil; 3 Department of Social Medicine, Federal University of Espirito Santo, Marechal Campos avenue, 1468 – Maruípe, Vitória, Espírito Santo CEP: 29040-090 Brazil

**Keywords:** Osteoporosis. Menopause. Body composition. Densitometry, Sarcopenia

## Abstract

**Background:**

The appendicular skeletal muscle mass index (ASMI) is an important risk indicator for osteoporosis because of the anatomical proximity and metabolic connection between muscle and bone mass. The present study investigated the relationship between ASMI and the bone mineral density (BMD) categories of postmenopausal women.

**Methods:**

In this cross-sectional study with a probabilistic sample, sociodemographic, lifestyle, menopause time, anthropometric, and physical activity variables were collected. ASMI and BMD were assessed by dual-energy X-ray absorptiometry (DXA). Participants were grouped according to BMD values into normal density, osteopenia, and osteoporosis. Multivariate logistic regression models were applied to verify the influence of ASMI on BMD. Data were analyzed using the SPSS statistical software, version 22. The significance level for all tests was set at 5%.

**Results:**

Of the 114 women analyzed, most were between 60 and 69.9 years of age (62.3%), on menopause for ≤19.0 (51.8%), self-declared brown race/color (49.1%), had < 4 years of education (41.2%), never smoked (69.0%) or drank alcohol (62.8%). Of these, 52.6% were classified as sufficiently active and 52.2% had regular sun exposure. Women with osteoporosis were older (*p* = 0.035), on menopause for a longer time (*p* = 0.011), underweight (*p* = 0.004), had adequate waist circumference (*p* = 0.017), and low ASMI values (*p* = 0.002). There was an association between the 1st tertile of ASMI and osteoporosis. However, after adjustments for age, race/color, and body mass index, the strength of association between BMD and ASMI was not maintained.

**Conclusions:**

ASMI was not associated with the BMD of the postmenopausal women evaluated. Total body and muscle mass, in addition to bone mass, should be monitored during menopause treatment. Longitudinal studies must be conducted to elucidate the mechanisms and gaps in this relationship.

## Background

Skeletal muscle mass (SMM) performs mechanical, structural, and metabolic functions in the human body [1]. Muscle mass is constantly changing from birth until it reaches its maximum peak at around 30 years of age. In adulthood, several factors interfere in the development of SMM, such as genetics, race/color, diet, physical activity, and hormone levels [2].

With increasing age, the musculoskeletal system progressively declines [3, 4], with consequent reduction in functional capacity and possible development of diseases such as osteoporosis and sarcopenia [3, 5].

The term sarcopenia was first used in 1989 by Irwin Rosenberg to refer to age-related loss of muscle mass and strength [6]. Years later, Baumgartner et al. [7] proposed the use of appendicular skeletal muscle mass (ASM) adjusted by height squared, called the appendicular skeletal muscle mass index (ASMI), as an indicator of low muscle mass [7, 8]. Since then, ASMI has been used as one of the main parameters to assess body muscle mass [3, 9].

In 2010, the European Working Group on Sarcopenia in Older People (EWGSOP) improved the concept of sarcopenia and recommended, in addition to low muscle mass, the use of low muscle strength or low physical performance for the diagnosis [10]. In its most recent review, the EWGSOP2 proposed an algorithm for the diagnosis of sarcopenia, followed by the assessment of muscle strength (pre-sarcopenia), low quantity or quality of muscle mass (sarcopenia), and impaired physical performance associated to the aforesaid parameters (severe sarcopenia) [3].

Although this aging-related musculoskeletal decline is common to all individuals [3, 6], it is accelerated in post-menopausal women, being mainly related to changes in the levels of hormones with important functions in bone and muscle health [11–14].

Regarding muscle mass, it has been suggested that low estrogen production, especially of the hormone estradiol, may favor sarcopenia [13, 14]. Through estrogen beta-receptors present in skeletal muscle, estradiol stimulates the activation and proliferation of satellite cells, promoting muscle repair. Reduced estrogen levels compromise this maintenance [14], in addition to being associated with an increase in pro-inflammatory cytokines that can degrade muscle proteins and reduce muscle regeneration capacity [11, 14].

Another mechanism involves the decrease in the hormone dehydroepiandrosterone (DHEA), which is related to the reduction in physical performance and muscle mass in women after menopause [15]. The decrease in testosterone levels after the first years of menopause is concomitant with the decrease in muscle mass, in which it plays an important role [11].

Regarding bone mass, estrogen regulates the coupling between bone resorption and formation [13]. With the reduction in estrogen production caused by menopause, osteoblasts produce an excess of the cytokine RANKL, which promotes osteoclastogenesis and bone resorption when bound to the RANK receptor [15, 16]. Estrogen deficiency reduces the secretion of osteoprotegerin (OPG), an inhibitor of RANKL secreted by osteoblasts after estrogen stimulation, thus increasing the activity of this cytokine. As a result, there is an increase in resorption at the expense of bone formation, which leads to skeletal disorder and deterioration in bone mineral density (BMD) [13, 16].

Bone mineral density is assessed using dual energy x-ray absorptiometry (DXA) and classified into normal BMD, osteopenia, osteoporosis, and osteoporosis associated with fracture risk, as established by the World Health Organization (WHO) [17].

Previous studies that evaluated the relationship between SMM and bone mass in postmenopausal women failed to clarify this relationship [7–14], producing conflicting results [18, 19]. These divergences may be related to the characteristics of the population and study design, methods for measuring and classifying SMM, as well as factors such as menopause time and muscle mass index, which can exert greater influence on bone mass when compared to SMM [15, 16].

Although SMM incites a growing interest, many studies use lean mass (muscles, tendons, and connective tissue) or total lean soft tissue (skeletal muscle mass and that of all other organs) as synonyms for SMM, what can affect the analysis of the aforementioned relationship [20, 21]. To investigate the influence of SMM on the preservation – or lack thereof – of bone mass, ASMI must be determined from the SMM obtained through dual-energy X-ray absorptiometry (DXA) [22].

Few studies analyzed the relationship between ASMI and BMD in postmenopausal women [4, 20, 23–29]. Therefore, starting from the premise that aging, associated with post-menopause, affects SMM negatively and potentializes the reduction of bone mass, in the present study we investigated the relationship between ASMI and the BMD categories of postmenopausal women.

## Methods

Cross-sectional observational study conducted on a probabilistic sample from the Climacteric and Osteoporosis Outpatient Clinics of a University Hospital in Vitória, Espírito Santo, Brazil, carried out from June 2019 to March 2020.

### Population

The study population consisted of postmenopausal women seen at a secondary-level public service. The inclusion criteria considered for this study were women aged between 55 and 85 years, in menopause for at least 12 months. Those under hormone replacement therapy, with cardiac implants, and who did not answer four telephone contact attempts were excluded.

### Sampling and sample drawing

The sample size was based on the number of consultations that took place in 2018 at the aforementioned clinics, which corresponded to 527 consultations. Duplicate consultations and women under 50 years of age (*n* = 185) were excluded, with 342 women remaining eligible for the study. A 95% confidence interval, a 5% margin of error, and a 21.3% prevalence of osteoporosis in women over 50 years of age were considered [30], resulting in a sample of 147 women.

The sample was selected through a simple random drawing using Excel® (*Office 2016*), allocated in a list, and drawn in a single step. Those who refused to participate in the study were replaced in a new drawing.

After this step, the women were invited to participate in the study via telephone contact.

### Instruments and study variables

Data were collected by properly trained and qualified professionals on the premises of the ELSA Investigation Center in the Espírito Santo state (IC-ES). A semi-structured questionnaire containing sociodemographic information (age in years, marital status, education and self-reported race/color). Self-declared race/color was defined as white, black, brown, yellow, and indigenous, as recommended by the Brazilian Institute of Geography and Statistics [31]. Lifestyle (smoking, alcohol consumption, level of physical activity, and sun exposure) and menopause time (years) were also evaluated. Women were classified as adult (< 60 years) and elderly (≥ 60 years) according to the WHO classification for developing countries [32].

Body mass and height were measured according to the method recommended by Lohman et al. [33]. BMI was calculated as the ratio between body mass (kg) and squared height (m^2^) and classified according to the WHO for adults [34] and to the recommendations of the Pan American Health Organization (PAHO) for the elderly [35].

Calf circumference (CC) was measured at the largest point of the calf, with the patient in the sitting position and knees flexed at 90 degrees [33]. Values ≤33 cm were classified as reduced [36]. Waist circumference (WC) was measured in duplicate at the midpoint between the last rib and the iliac crest. Values ≥80 cm were classified as high [34].

Handgrip strength (HGS) was assessed both in the dominant hand (DHGS) and in the non-dominant hand (NDHGS) through the method recommended by the American Society of Hand Therapists (ASHT) [37]. The process was conducted in triplicate with 1-min interval between measurements: maximum force was applied for 5 s while the individual evaluated was verbally encouraged. The maximum value was considered for analysis. The cutoff point adopted was < 16.0 kg, as proposed by the European Working Group on Sarcopenia in Older People (EWGSOP2) [3].

The Timed Get-Up-and-Go test (TUG) was performed according to the method proposed by Podsiadlo and Richardson [38]. The test was performed in triplicate using the mean time value and adopting a cutoff point of > 20 s [3].

To obtain ASMI, the appendicular skeletal muscle mass (ASM) was first estimated through Dual-Energy X-ray Absorptiometry (DXA) (GE Lunar Prodigy Advance®), using the GE Encore software, version 14.10, properly calibrated and configured to use the reference database of the National Health and Nutrition Examination Survey [39]. For the examination, women fasted for 4 h beforehand, wore only a gown, and met the recommendations of the International Society for Clinical Densitometry [40]. To minimize interobserver variation, all body densitometry examinations were performed only by a properly trained, certified radiology technician and were interpreted and signed by a single physician. Subsequently, ASMI was calculated as the ratio between ASM (kg) and squared height (m^2^) [3]. Values were classified according to the tertiles themselves.

BMD was also identified by DXA and classified as normal BMD (T-score ≥ − 1 DP), osteopenia (T-score between − 1 and − 2.5 DP), and osteoporosis (T-score ≤ − 2.5 DP), according to the cutoff points recommended by the WHO [17]. The proximal femur and lumbar spine (L1-L4) were evaluated for this diagnosis. Values were obtained from the medical record and confirmed with the physician in charge.

The level of physical activity (PA) was estimated using the International Physical Activity Questionnaire (IPAQ), validated for the Brazilian population in its long version [41]. To avoid the overestimation of PA levels, only the sum of questions related to leisure and transportation was considered [42]. Women who reported performing 150 min or more of weekly PA were classified as sufficiently active, while those deemed insufficiently active did not reach the level recommended by the WHO [43]. The participants were also asked about sun exposure habits and their answers dichotomized into yes or no.

### Ethical aspects

The study complied with the Resolution CNS 466/12 of the Ministry of Health/Brazil [44] and was approved by the Research Ethics Committee of the Federal University of Espírito Santo under CAAE: 88131818.0.0000.5060 and protocol #: 2.621.794.

### Statistical analysis

Descriptive analysis was expressed as percentage for categorical variables. For data analysis, the overweight and obese categories were categorized as overweight. Fisher’s exact and chi-square tests were applied to verify the difference between proportions according to the BMD categories. Multivariate logistic regression models were adjusted considering two main outcomes: osteopenia and osteoporosis. All variables that presented *p* values < 0.10 in the bivariate associations were included in the models. Collinear or strongly correlated variables were not included in the models. Those with values of *p* < 0.05 in the univariate analysis were kept in the final model. Odds ratio and the respective confidence intervals were calculated. Data were analyzed using the software SPSS 22.0 and the significance level adopted for all tests set at 5.0%.

## Results

**Fig. 1:**
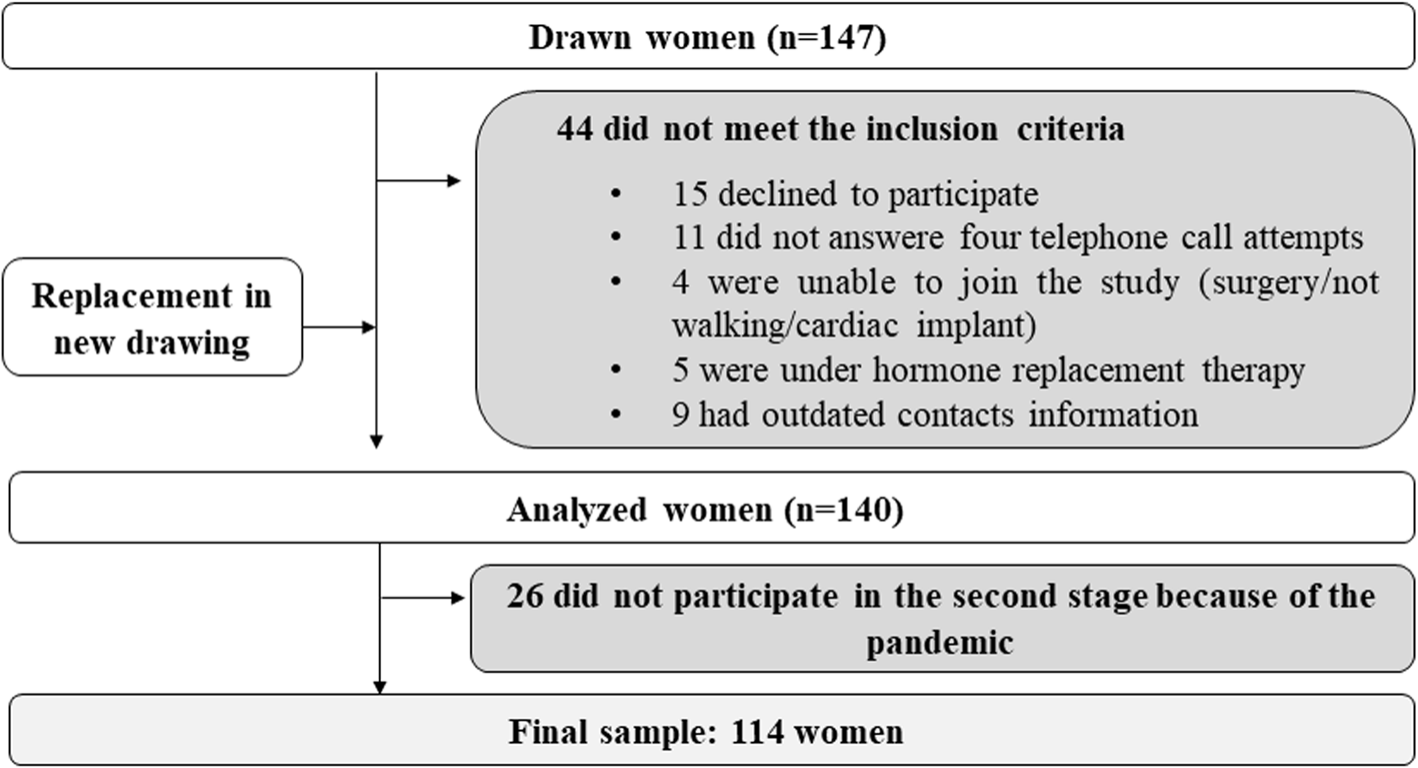
Selection Flowchart

In the final stage of sample selection, 44 women did not meet the inclusion criteria and, therefore, other women were drawn and included in the sample. After the final selection, 140 women were analyzed. However, the collection was interrupted due to the advance of the pandemic by the new coronavirus (SARS-CoV2), mainly because the study included a population at risk, thus our final sample included 114 women (Fig. 1).

Sociodemographic, lifestyle, and BMD variables are described in Table 1. There was a predominance of women aged between 60 and 69.9 years (62.3%), on menopause for ≤19.0 years (51.8%), self-declared brown race/color (49.1%), with less than four years of education (41.2%), and who had never smoked (69.0%) or consumed alcohol (62.8%). Of these, 52.6% were classified as sufficiently active and 52.2% had regular sun exposure (Table 1).

**Table 1:** Distribution of sociodemographic and lifestyle variables according to bone mineral density (BMD) of postmenopausal women

Variables	Total	BMD			p value
		Normal (***n*** = 27; 23.7%)	Osteopenia (***n*** = 48; 42.1%)	Osteoporosis (***n*** = 39; 34.2%)	
**Age group (years)**^**a**^					**0.035**
50.0–59.9	15 (13.2)	7 (46.7)	7 (46.7)	1 (6.7)	
60.0–69.9	71 (62.3)	17 (23.9)	28 (39.4)	26 (36.6)	
≥ 70.0	28 (24.6)	3 (10.7)	13 (46.4)	12 (42.9)	
**Menopause time (years)**^**b**^					**0.011**
≤ 19.0	59 (51.8)	20 (33.9)	25 (42.4)	14 (23.7)	
> 19.0	55 (48.2)	7 (12.7)	23 (41.8)	25 (45.5)	
**Race/color**^**b**^					0.087
White	43 (37.7)	10 (46.5)	13 (30.2)	20 (46.5)	
Black	9 (7.9)	2 (22.2)	4 (44.4)	3 (33.3)	
Yellow	6 (5.3)	2 (33.3)	2 (33.3)	2 (33.3)	
Brown	56 (49.1)	13 (23.2)	29 (51.8)	14 (25.0)	
**Schooling (years)**^**b**^					0.331
< 4	47 (41.2)	9 (19.1)	21(44.7)	17 (36.2)	
4–8	32 (28.1)	10 (31.3)	9 (28.1)	13 (40.6)	
> 8	35 (30.7)	8 (22.9)	18 (51.4)	9 (25.7)	
Smoking*^a^					0.937
Never smoked	78 (69.0)	18 (23.1)	33 (42.3)	27 (34.6)	
Smokes	6 (5.3)	1 (16.7)	2 (33.3)	3 (50.0)	
Used to smoke	29 (25.7)	8 (27.6)	12 (41.4)	9 (31.0)	
Alcohol intake*^a^					0.549
Never drank	71 (62.8)	18 (25.4)	27 (38.0)	26 (36.6)	
Drinks	15 (13.3)	4 (26.7)	7 (46.7)	4 (26.7)	
Used to drink	27 (23.9)	5 (18.5)	13 (48.1)	9 (33.3)	
Physical activity level*^b^					0.380
Insufficiently active	53 (46.5)	15 (28.3)	23 (43.4)	15 (28.3)	
Sufficiently active	60 (52.6)	12 (20.0)	24 (40.0)	24 (40.0)	
Sun exposure**^b^					0.451
Yes	58 (52.2)	14 (24.1)	21 (36.2)	23 (39.7)	
No	53 (47.8)	12 (22.6)	25 (47.2)	16 (30.2)	

*n = 113; **n = 111; BMD: bone mineral density; ^a^Fisher’s exact test; ^b^Chi-square test. Bold: *p* value < 0.05

BMD categories were significantly different in different age groups and menopause times (*p* < 0.05) (Table 1). Osteoporosis was more prevalent among women aged ≥70.0 years (*p* = 0.035) and those on menopause for over 19 years (*p* = 0.011).

When evaluating the distribution of anthropometric, body composition, HGS, and TUG variables within the BMD categories, we observed that osteoporosis was significantly more frequent in women with ASMI classified in the lowest tertile (*p* = 0.002), underweight (*p* = 0.004), and adequate WC (*p* = 0.017) (Table 2).

**Table 2:** Distribution of anthropometric, body composition, handgrip strength, and Timed Get Up and Go test variables according to bone mineral density (BMD) categories in postmenopausal women

Variables	Total	BMD			p value
		Normal (n = 27 23.7%)	Osteopenia (n = 48 42.1%)	Osteoporosis (n = 39 34.2%)	
**ASMI (m**^**2**^**)**^**a**^	n (%)				**0.002**
1st tertile (5.68 ± 0.31)	38 (33.3)	2 (5.3)	16 (42.1)	20 (52.6)	
2nd tertile (6.72 ± 0.28)	38 (33.3)	14 (36.8)	12 (31.6)	12 (31.6)	
3rd tertile (7.78 ± 0.53)	38 (33.3)	11 (28.9)	20 (52.6)	7 (18.4)	
**BMI**^**b**^					**0.004**
Underweight	20 (16.7)	1 (5.0)	7 (35.0)	12 (60.0)	
Eutrophic	43 (38.6)	9 (20.9)	16 (37.2)	18 (41.9)	
Overweight	51 (44.7)	17 (33.3)	25 (49.0)	9 (17.6)	
**CC**^**b**^					0.186
Adequate	98 (86.0)	26 (26.5)	39 (39.8)	33 (33.7)	
Reduced	16 (14.0)	1 (6.3)	9 (56.3)	6 (37.5)	
**WC**^**b**^					**0.017**
Adequate	13 (11.4)	1 (7.7)	3 (23.1)	9 (69.2)	
Elevated	101 (88.6)	26 (25.7)	45 (44.6)	30(29.7)	
**DHGS**^**b**^					0.250
Adequate	107 (93.9)	25 (23.4)	47 (43.9)	35 (32.7)	
Reduced	7 (6.1)	2 (28.6)	1 (14.3)	4 (57.1)	
**NDHGS**^**b**^					0.546
Adequate	100 (87.7)	23 (23.0)	44 (44.0)	33 (33.0)	
Reduced	14 (12.3)	4 (28.6)	4 (28.6)	6 (42.9)	
**TUG (s)**^**b**^					1.000
Adequate	111 (97.4)	26 (23.4)	47 (42.3)	38 (34.2)	
Inadequate	3 (2.6)	1 (33.3)	1 (33.3)	1 (33.3)	

*ASMI* Appendicular Skeletal Muscle Mass Index, *BMI* Body Mass Index, *CC* Calf Circumference,m *WC* Waist circumference, *HC* Hip Circumference, *DHGS* Dominant handgrip strength, *NDHGS* Non-Dominant Handgrip Strength, *TUG* Timed Get Up and Go test. ^a^Chi-square test; ^b^Fisher’s exact test. Bold: *p* value < 0.05

**Fig. 2:**
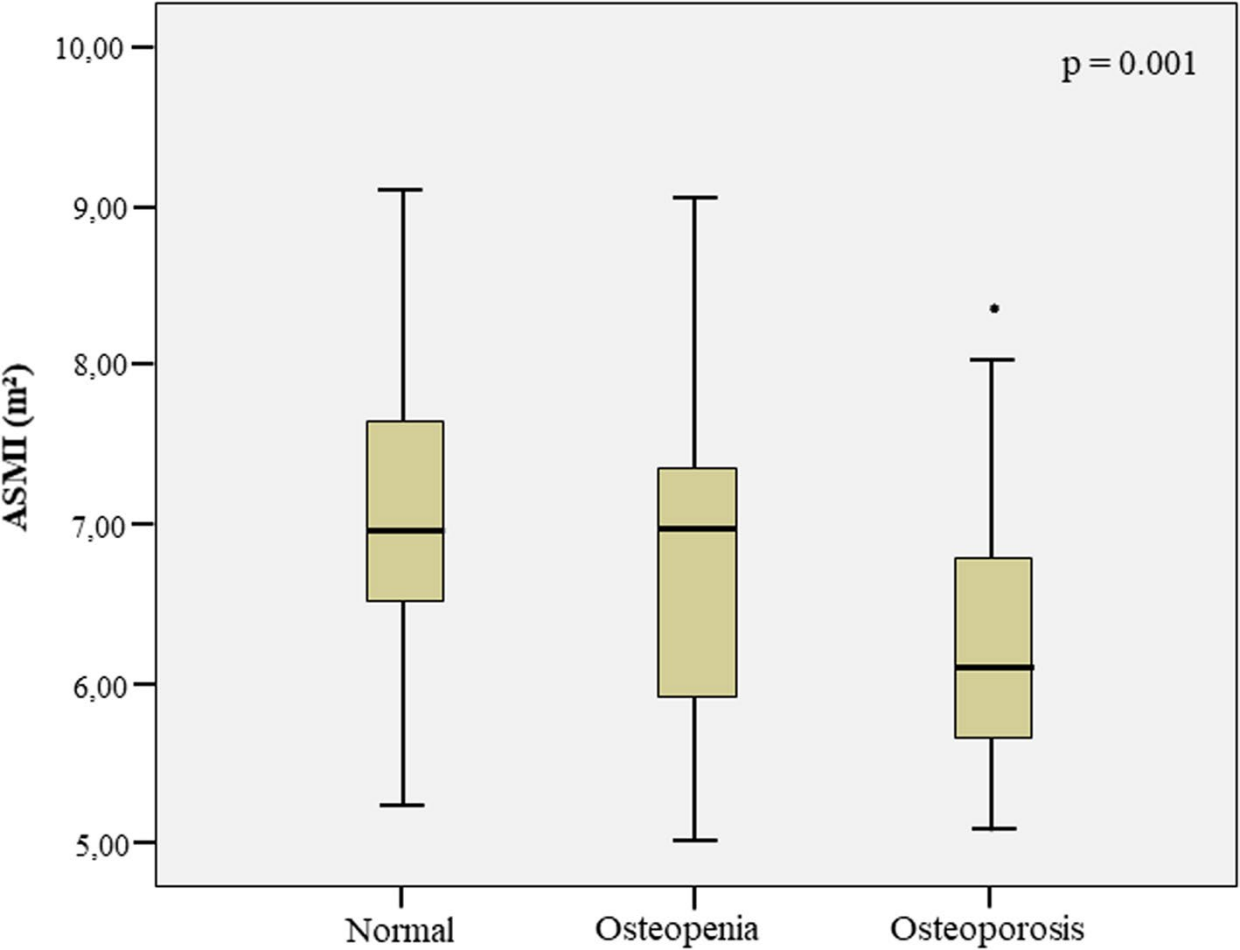
Boxplot of the Appendicular Skeletal Muscle Mass Index (ASMI) according to bone mineral density (BMD) categories. *Difference observed between women with normal BMD and osteoporosis

Figure 2 shows a significant difference between ASMI mean values according to the BMD categories. The average ASMI of the normal BMD group was 7.78 m^2^, while in the osteopenia and osteoporosis groups it was 6.72 m^2^ and 5.68 m^2^, respectively. Differences were observed between the osteoporosis group and the normal BMD and osteopenia groups (*p* = 0.001).

After adjustments for age group and race/color (model 1), we observed that the 1st tertile of the ASMI remained associated with osteoporosis (OR: 10.45 [CI 95%: 1.74–62.7]). However, after adjusting for age group, race/color, and BMI (model 2), the strength of association between ASMI and BMD was not maintained (Table 3).

**Table 3:** Logistic regression models for the appendicular skeletal muscle mass index (ASMI) according to bone mineral density (BMD) in postmenopausal women

	OSTEOPENIA N = 48			OSTEOPOROSIS N = 39		
	Raw OR (CI 95%)	Model 1 OR (CI 95%)	Model 2 OR (CI 95%)	Raw OR (CI 95%)	Model 1 OR (CI 95%)	Model 2 OR (CI 95%)
**ASMI (m**^**2**^**)**						
1st Tertile	4.40 (0.85–22.76)	3.25 (0.60–17.52)	3.42 (0.42–27.6)	**15.7 (2.77–89.1**	**10.45 (1.74–62.7)**	3.75 (0.40–35.0)
2nd Tertile	0.47 (0.16–1.37)	0.37 (0.12–1.16)	0.40 (0.12–1.35)	1.34 (0.40–4.57)	0.91 (0.16–1.37)	0.67 (0.15–2.88)
3rd Tertile	1	1	1	1	1	1

*OR* Odds Ratio. Model 1: adjusted for age group and race/color. Model 2: adjusted for age group, race/color, and body mass index. Bold: *p* value < 0.05. Normal BMD: reference

## Discussion

In this study, ASMI did not remain associated with the BMD of postmenopausal women after the proposed adjustments. The women evaluated showed satisfactory lifestyle habits: they did not smoke or take alcohol, were physically active, and had preserved muscle mass and functional capacity, albeit associated with a predominance of excess weight and central adiposity. These lifestyle and nutritional status characteristics may have influenced the results obtained, as they act directly in the preservation of bone and muscle mass [15, 45–48].

The relationship between ASMI or SMM and BMD, although widely discussed, is still uncertain [49]. The results of this study differ from data available in the literature that suggest SMM influences bone mass proportionally. Some studies state that this association reflect not only the effect of mechanical muscle load on bone tissue, through muscle contraction, but also the functional relationship between these two systems [4, 25, 29]. On the other hand, other investigations found no association between ASMI and BMD, corroborating our results [24, 27, 50–53].

Some hypotheses suggest that factors such as excess weight and central adiposity may influence BMD through different mechanisms, such as adaptation of the bone structure to support the body’s adipose tissue mass [54]. Another mechanism would be the increase in the aromatization of androgens into estrogen in the adipose tissue, thus stimulating bone formation in women after menopause [51, 55].

These inconclusive results are in great part attributed to the use of different methods to measure muscle mass, added to the heterogeneity of the DXA-measured ASMI nomenclature or even the lack of consensus in the literature to define ASMI and its cutoff points, making it difficult to compare and discuss the results [21, 51, 56, 57].

Due to the lack of Brazilian references, the EGWSOP2 proposal was used to define ASMI [3] which has been considered the most recommended to define muscle mass depletion [22], in addition to demonstrating greater associations with negative clinical outcomes [3, 23, 57, 58]. Furthermore, lean mass, which is composed of bone, skin, and muscle, is misused as SMM, what can result in confusing conclusions about the influence of SMM on BMD [21]. Finally, the individuals total body mass and body composition may also influence the bone-muscle relationship [19, 27].

According to Saarelainen et al. [51], who evaluated 198 Finnish postmenopausal women, excess weight and fat distribution affect the measurement of muscle mass by overestimating BMD, making it important to control these components. This fact may explain the results obtained here, since the women evaluated were overweight and had abdominal fat accumulation. This explanation can also be supported by the fact that after adjustment for BMI in the final regression models, the association between ASMI and BMD was lost. In this group, BMI seems to have influenced BMD more than ASMI, much like previously reported outcomes [59, 60].

As to the possibility that bone mass increases or decreases linearly based on BMI values [51], here we observed that the higher prevalence of osteoporosis was associated with low weight according to BMI, thus confirming this possibility. Low weight is considered a risk factor for the development of osteoporosis [19, 46, 61]. A population-based meta-analysis that evaluated BMI as a predictor of fracture risk showed that low weight confers a marked risk for all osteoporotic fractures, regardless of age and sex, but dependent on BMD [61].

Mazocco and Chagas [59], when evaluating 393 Brazilian postmenopausal women, identified a lower prevalence of osteopenia and osteoporosis among obese women. Likewise, Shayganfar et al. [19] observed a significant increase in BMD with an increase in BMI in 1056 postmenopausal women. However, although some studies support the beneficial effect of excess weight on BMD, it is important to emphasize that obesity is a risk factor for different comorbidities [62]. Both low weight and obesity can determine the intensification of osteoporosis and adversely affect the function and quality of SMM in postmenopausal women, in addition to their general health and quality of life [55, 60, 62, 63]..

The investigation and clarification that between of the influence that SMM plays on bone mass in postmenopausal women is a challenge, given the many variables that can affect these tissues and need to be controlled and included in future studies, such as serum hormonal levels and the use of protein supplementation.

This study has as a limitation not being representative of the general population of postmenopausal women, as it was carried out with a single group of women seen at a secondary level outpatient clinic of a public health service, thus preventing extrapolation of the results. On the other hand, as a contribution, this study is one of the few investigating the influence of ASMI on the BMD of postmenopausal women in outpatient care, using a reference standard for the analysis of muscle mass and BMD, in addition to validated questionnaires. In practical terms, the reported outcomes are clinically valid. We believe longitudinal studies should be conducted to elucidate the mechanisms and gaps in the relationship between ASMI and BMD.

The results of this study demonstrate the importance of monitoring body mass during menopause treatment, prioritizing the preservation of muscle mass and reduction of bone mass depletion. Therefore, the adoption of healthy lifestyle habits should be encouraged, including the practice of physical activity and dietary intervention, such as an adequate protein intake or supplementation when necessary.

## Conclusion

ASMI was not associated with BMD in the postmenopausal women evaluated. Among women with osteoporosis, there was a higher prevalence of those aged over 70 years, on menopause for a longer time, and with adequate WC, in addition to women with lower ASMI averages and classified as underweight. This study demonstrates the importance of tracking total body and muscle mass, as well as bone mass during menopause treatment.

## Data Availability

The data that support the findings of this study are available from the corresponding author on reasonable request.

## Abbreviations

ASMI: Appendicular Skeletal Muscle Mass Index
BMD: Bone Mineral Density
SMM: Skeletal Muscle Mass
BMI: Body Mass Index
CC: Calf circumference
WC: Waist circumference
HC: Perímetro do Quadril. Hip circumference
DHGS: Dominant Handgrip Strength
NDHGS: Non-dominant Handgrip Strength
TUG: Timed Get-Up-and-Go test
OR: Odds Ratio
PA: Physical Activity
WHO: World Health Organization
PAHO: Pan American Health Organization
ASHT: American Society of Hand Therapists
IPAQ: International Physical Activity Questionnaire
DXA: Dual Energy X-ray Absorptiometry
CI- ES: ELSA Investigation Center in the Espírito Santo state
